# Targeting the AMPK Pathway with Natural Products for Heart Failure: A Systematic Review of Preclinical Evidence

**DOI:** 10.3390/biomedicines14040765

**Published:** 2026-03-27

**Authors:** Xiaoxiao Huang, Haitong Wan

**Affiliations:** Academy of Chinese Medical Sciences, Henan University of Chinese Medicine, Zhengzhou 450046, China; hxx_sweet@163.com

**Keywords:** heart failure, AMPK signaling pathway, traditional Chinese medicine, systematic review

## Abstract

**Background**: Heart failure (HF) is a leading cause of morbidity and mortality worldwide. AMP-activated protein kinase (AMPK) is a central regulator of energy homeostasis, and its dysregulation is implicated in HF pathophysiology. Traditional Chinese Medicine (TCM) has been investigated in HF management, but a systematic synthesis of preclinical evidence on TCM-mediated AMPK modulation is lacking. **Methods**: PubMed and Web of Science were searched from January 2020 to December 2025 using a comprehensive strategy combining terms for AMPK, HF, and TCM. Studies were included if they were original research investigating TCM-derived compounds or formulas in HF models and reporting AMPK modulation. Study quality and evidence levels were assessed using predefined criteria. The review was conducted in accordance with PRISMA 2020 guidelines. **Results**: Of 243 records identified, 56 studies met the inclusion criteria (7 from database search and 49 from manual screening). Direct evidence for AMPK-dependent cardioprotection was limited. Cinnamaldehyde and paeoniflorin showed the most rigorous validation with confirmed target engagement and loss-of-function rescue. Berberine, crocin, ginsenoside Rb1, and honokiol demonstrated pathway-specific effects validated by pharmacological or genetic approaches. Most complex herbal formulas provided correlative evidence only, with Fuyu Decoction being a notable exception where AMPK agonist EX229 confirmed pathway involvement. **Conclusions**: Current evidence for TCM-mediated AMPK modulation in HF remains predominantly preliminary and correlative. Future research should prioritize causality validation using genetic models and human-relevant systems.

## 1. Introduction

Heart failure (HF) is a complex clinical syndrome characterized by structural or functional cardiac impairment that compromises ventricular filling or ejection of blood, affecting an estimated 64 million people globally [1,2,3]. Despite advances in guideline-directed medical therapy, HF remains a progressive syndrome with high morbidity and mortality [4]. Adenosine monophosphate-activated protein kinase (AMPK) functions as a crucial cellular energy sensor and metabolic regulator essential for maintaining cardiomyocyte energy homeostasis [5]. Dysregulation of AMPK signaling is intimately associated with core pathophysiological mechanisms driving HF, including energy depletion, oxidative stress, inflammation, apoptosis, autophagy dysregulation, and fibrosis.

Traditional Chinese Medicine (TCM), rooted in the principles of holistic health and syndrome differentiation, has a long history of application in HF management. Therapeutic strategies such as “tonifying qi, warming yang, activating blood circulation, and promoting diuresis” have demonstrated clinical advantages. Modern pharmacological studies reveal that active constituents and compound formulations of Chinese herbal medicine can modulate multiple pathways, with AMPK emerging as a potential molecular target.

Previous reviews have separately addressed either the role of AMPK in HF pathogenesis or the general cardioprotective effects of TCM. However, a systematic synthesis of preclinical evidence specifically focused on TCM-mediated AMPK modulation in HF, with critical appraisal of mechanistic strength and evidence quality, is currently lacking. This systematic review aims to (1) systematically identify and synthesize available preclinical evidence on TCM interventions targeting AMPK in HF; (2) critically appraise the mechanistic strength and quality of evidence; (3) distinguish validated direct AMPK mechanisms from correlative findings and unproven hypotheses; and (4) identify research gaps to guide future investigations.

## 2. Methods

### 2.1. Search Strategy

We systematically searched PubMed and Web of Science databases for articles published between January 2020 and December 2025. The following search strategy was used in PubMed to ensure comprehensive and reproducible results: (“AMP-Activated Protein Kinases” OR “AMPK” OR “AMP-activated protein kinase”) AND (“Heart Failure” [Mesh] OR “cardiomyopathy” OR “cardiac dysfunction” OR “myocardial failure”) AND (“natural products” OR “herbal medicine” OR “traditional Chinese medicine” OR “phytochemicals”) The same search strategy was adapted for Web of Science using equivalent keywords and topic searches. Additionally, we performed a manual search of reference lists of relevant articles and reviews to identify additional studies.

### 2.2. Software and Tools

As this study is a systematic review, no experimental equipment or materials were used. Reference management, data extraction and synthesis, and the creation of schematic illustrations were performed using EndNote 2025 (Clarivate Analytics, Philadelphia, PA, USA), Microsoft Excel (Microsoft Corporation, Redmond, WA, USA), and BioRender (BioRender Inc., Toronto, ON, Canada), respectively.

### 2.3. Inclusion and Exclusion Criteria

Studies were included if they met the following criteria: (1) Peer-reviewed original research articles published in English. (2) Investigated the effects of TCM-derived compounds or herbal formulas in heart failure or relevant cardiac disease models (e.g., myocardial infarction, pressure overload, cardiomyopathy, toxin-induced cardiac injury). (3) Reported modulation of the AMPK pathway as either a primary or secondary mechanistic finding. Studies were excluded if they (1) Were conference abstracts, case reports, editorials, letters, or non-peer-reviewed publications. (2) Did not involve a heart failure or cardiac disease model (e.g., studies limited to non-cardiac cells, healthy animals without cardiac stress, or non-cardiac diseases). (3) Did not assess AMPK signaling.

### 2.4. Study Selection and Data Extraction

Two independent reviewers screened titles and abstracts of all retrieved records. Full texts of potentially eligible articles were then assessed independently by the same reviewers. Any disagreements were resolved through discussion. Data were extracted using a standardized form, including: first author, year, TCM intervention, experimental model, HF type, AMPK-related findings, and any validation methods employed.

### 2.5. Evidence Quality Assessment

To assess methodological rigor and enable reproducible assessment, we evaluated whether each study employed: (1) pharmacological AMPK modulators (e.g., Compound C, AICAR); (2) genetic manipulation (e.g., siRNA, knockout models); (3) direct binding assays (e.g., molecular docking, CETSA). Based on this, evidence was categorized into three tiers: direct AMPK mechanism (confirmed by loss-of-function approaches or direct binding assays), correlative evidence (AMPK changes shown without pathway-specific validation), and hypothesized/unproven (AMPK involvement inferred from indirect observations or network pharmacology predictions).

### 2.6. PRISMA Guideline Statement

**Figure 1:**
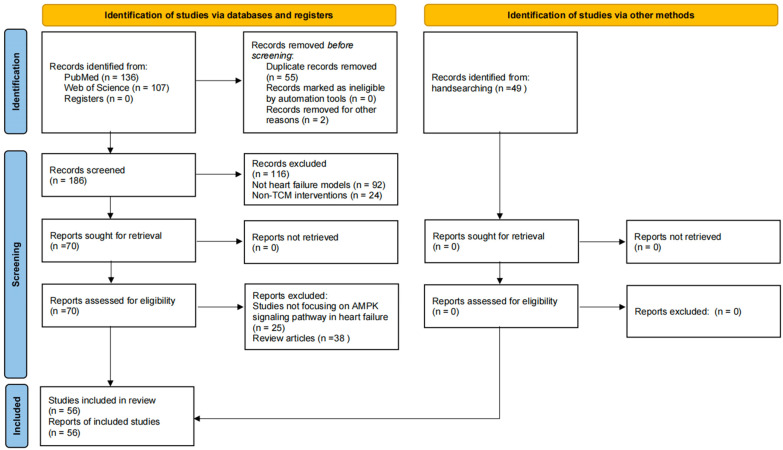
The literature search and screening flowchart.

This systematic review was conducted and reported in accordance with the Preferred Reporting Items for Systematic Reviews and Meta-Analyses (PRISMA) 2020 statement [6]. The PRISMA 2020 checklist is provided as Supplementary Materials. The review protocol was registered in PROSPERO (CRD420261341794). The study selection process is summarized in Figure 1.

## 3. Results

### 3.1. Study Selection

A total of 243 records were identified from PubMed (*n* = 136) and Web of Science (*n* = 107). After removing 55 duplicates and 2 retracted articles, 186 unique records were screened based on titles and abstracts. Of these, 116 records were excluded, including 92 that did not involve heart failure models and 24 with non-TCM interventions. The remaining 70 full-text articles were sought for retrieval, and all were successfully obtained. Following full-text assessment, 25 studies were excluded because they did not focus on the AMPK signaling pathway in heart failure, and 38 were review articles, leaving 7 studies from the database search. Additionally, 49 studies were identified through handsearching. In total, 56 studies met the inclusion criteria and were included in this review.

### 3.2. Study Characteristics

The 56 included studies comprised investigations of both active compounds (*n* = 6) and complex herbal formulas (*n* = 6). Study models included pressure overload-induced HF, myocardial infarction-induced HF, isoproterenol-induced HF, adriamycin-induced chronic HF, and HF with preserved ejection fraction (HFpEF). Detailed characteristics of included studies are presented in Table 1 and Table 2.

### 3.3. AMPK Pathway in Heart Failure

#### 3.3.1. Structure, Activation, and Physiological Functions of the AMPK Pathway

Adenosine monophosphate-activated protein kinase (AMPK) is an evolutionarily conserved energy-sensing kinase that is ubiquitously expressed in eukaryotic cells. It functions as a heterotrimeric complex composed of a catalytic α subunit (α1 or α2), a scaffolding β subunit (β1 or β2), and a regulatory γ subunit (γ1, γ2, or γ3) [7]. In cardiac tissue, the AMPKα2 isoform is predominant and functionally distinct from α1: α2 primarily regulates myocardial energy metabolism, mitochondrial homeostasis, and autophagy, whereas α1 is more involved in modulating anti-inflammatory responses and cell survival pathways [8]. The α subunit contains the core kinase domain, and phosphorylation at a conserved threonine residue (Thr172) within this domain constitutes the central step for AMPK activation, directly determining the efficiency of downstream signal transduction. The β subunit anchors the complex and interacts with glycogen via its carbohydrate-binding module (CBM), thereby linking AMPK to glycogen metabolism and cardiac-specific functional modulation [9]. The γ subunit contains four cystathionine β-synthase (CBS) repeats that competitively bind AMP, ADP, or ATP. Binding of AMP or ADP promotes AMPK activation, whereas ATP binding inhibits it [8]. This AMP/ADP/ATP binding ability enables the complex to sense fluctuations in cellular energy status and transduce them into a biochemical signal.

**Figure 2:**
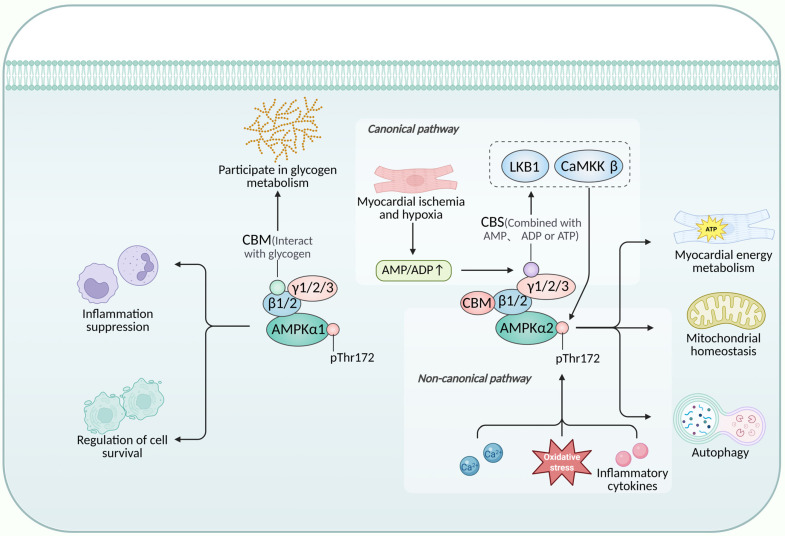
Schematic illustration of AMPK structure, activation pathways, and isoform-specific functions in cardiomyocytes. For detailed description, see Section 3.3.1. Created in BioRender. Peng, S. (2026) https://BioRender.com/0u9olfy (accessed on 13 March 2026).

AMPK activation occurs through both canonical and non-canonical mechanisms. In the canonical pathway, energy stress such as myocardial ischemia elevates intracellular AMP/ADP, promoting Thr172 phosphorylation by upstream kinases LKB1 or CaMKKβ [10]. Non-canonical pathways operate independently of AMP/ADP accumulation and are mediated by signals such as calcium flux, oxidative stress, or inflammatory cytokines [11,12]. A schematic overview is provided in Figure 2.

#### 3.3.2. Dysregulation of the AMPK Pathway and Its Cardioprotective Mechanisms in Heart Failure

**Figure 3:**
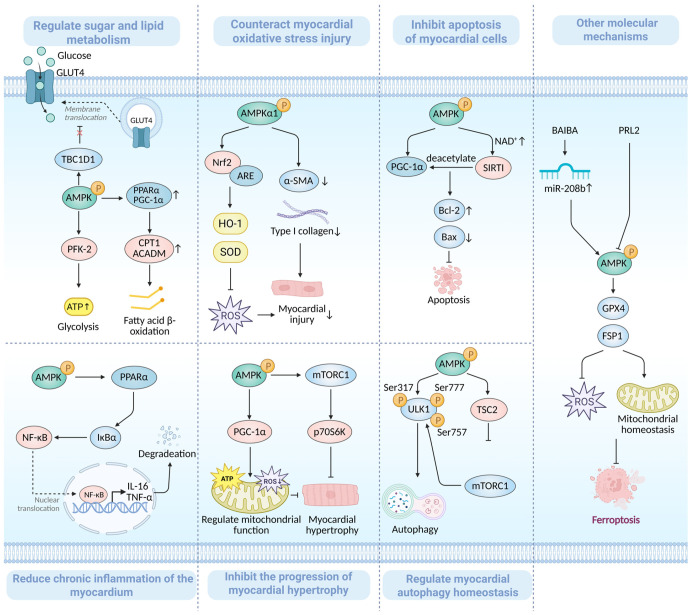
Schematic illustration of AMPK pathway-mediated cardioprotective mechanisms in heart failure. For detailed description, see Section 3.3.2. Created in BioRender. Peng, S. (2026) https://BioRender.com/0u9olfy (accessed on 13 March 2026).

Building upon the structural and physiological foundation of AMPK outlined above, this section examines its critical role in the pathogenesis of heart failure. Under pathological conditions such as myocardial ischemia and pressure overload, endogenous AMPK activation is often insufficient to meet the elevated metabolic demand. This insufficiency compromises the regulation of myocardial energy metabolism, exacerbating metabolic imbalance and energy deprivation [13]. Therefore, targeted activation of the AMPK pathway may represent a therapeutic strategy for HF, aimed at restoring myocardial energy homeostasis and modulating core pathogenic mechanisms—such as cardiomyocyte apoptosis, oxidative stress, and cardiac fibrosis [14]. The cardioprotective roles of AMPK in heart failure are illustrated in Figure 3.

Regulating Glucose and Lipid Metabolism to Maintain Myocardial Energy Homeostasis. Cardiomyocytes exhibit high energy demands that are met primarily through the continuous oxidation of glucose and fatty acids, a process fundamental to sustaining normal cardiac contractile function [15]. In heart failure, however, energy metabolic dysregulation becomes a prominent pathological feature, characterized by reduced glucose uptake, impaired glycolysis, and diminished fatty acid oxidation [16]. Glucose transporter type 4 (GLUT4) is the principal mediator of glucose uptake in cardiomyocytes. Under basal conditions, GLUT4 is sequestered within intracellular vesicles; in response to energy stress or insulin signaling, these vesicles rapidly translocate to the plasma membrane to enhance glucose import [17]. TBC1 domain family member 1 (TBC1D1) acts as a negative regulator by binding to GLUT4 vesicles and inhibiting their translocation. Studies in a cardiomyopathy model have shown that AMPK can phosphorylate TBC1D1 to relieve this inhibition [18]. This mechanism is likely conserved in the failing heart, where activated AMPK promotes GLUT4 membrane translocation to increase glucose uptake. In addition, AMPK promotes glycolytic flux by activating phosphofructokinase-2/fructose-2,6-bisphosphatase (PFK-2), which elevates fructose-2,6-bisphosphate (F2,6BP) levels, a key allosteric activator of glycolysis. This accelerates glycolytic ATP production, contributing to the maintenance of myocardial energy homeostasis under conditions of chronic metabolic stress [19]. Beyond glucose metabolism, AMPK cooperates with the peroxisome proliferator-activated receptor alpha (PPARα) pathway to regulate cardiac fatty acid oxidation [20]. Zhang et al. [21] demonstrated in a rabbit model of chronic heart failure (CHF) that AMPK activation upregulates PPARα and PGC-1α expression, leading to increased levels of carnitine palmitoyltransferase 1 (CPT-1) and medium-chain acyl-CoA dehydrogenase (ACADM). This coordinated regulation restores fatty acid uptake, transport, and β-oxidation, thereby improving myocardial energy metabolism. By enhancing GLUT4-mediated glucose uptake and PFK-2-driven glycolysis while optimizing fatty acid oxidation via the PPARα pathway, AMPK helps restore myocardial mitochondrial homeostasis and counteract the energy metabolic dysregulation central to HF progression.

Counteracting Myocardial Oxidative Stress Injury. Oxidative stress, characterized by excessive accumulation of reactive oxygen species (ROS) and dysfunction of the endogenous antioxidant defense system, contributes significantly to cardiomyocyte injury by inducing lipid peroxidation, protein denaturation, and DNA damage [22]. The transcription factor nuclear factor erythroid 2-related factor 2 (Nrf2) serves as a master regulator of the cellular antioxidant response. Upon activation, Nrf2 translocates to the nucleus and binds to antioxidant response elements (AREs), thereby driving the expression of cytoprotective enzymes such as heme oxygenase-1 (HO-1) and superoxide dismutase (SOD), which collectively enhance the cellular capacity to neutralize ROS [23,24]. Accumulating evidence implicates excessive ROS-driven oxidative stress as a key contributor to the progression of myocardial damage in heart failure [25]. During HF progression, AMPK activation acts as an endogenous compensatory mechanism that protects the myocardium from oxidative injury. Myocardial ischemia/reperfusion (I/R) injury, a common event following cardiovascular procedures, often precipitates or exacerbates HF. Hou et al. [26] demonstrated in a myocardial I/R model that activation of the AMPKα1/Nrf2 signaling axis significantly upregulated the expression of Nrf2 and its downstream target HO-1. This activation concurrently suppressed endoplasmic reticulum stress-mediated apoptosis and reduced the expression of profibrotic markers, including α-smooth muscle actin (α-SMA) and type I collagen. These findings illustrate the coordinated role of AMPK in alleviating oxidative stress, inhibiting apoptosis, and attenuating fibrosis. Therefore, pharmacological or physiological activation of AMPK may help mitigate oxidative stress-induced myocardial damage in HF.

Inhibiting Excessive Cardiomyocyte Apoptosis. Cardiomyocyte apoptosis is a pivotal pathological event in heart failure, resulting in the irreversible loss of functional contractile units. In HF, the myocardium is subjected to a sustained pathological microenvironment characterized by ischemia/hypoxia, oxidative stress, and chronic inflammation. These stimuli disrupt the delicate regulatory balance of apoptosis, promoting its excessive activation. Cardiomyocytes are terminally differentiated cells with minimal regenerative capacity; therefore, their loss through apoptosis directly reduces the number of functional contractile cells and impairs systolic function. Furthermore, apoptotic cells release damage-associated molecular patterns that promote interstitial fibrosis, exacerbating adverse ventricular remodeling and creating a self-perpetuating cycle of progressive cell loss and cardiac dysfunction [27,28]. AMPK activation counteracts apoptosis through multiple mechanisms. In a mouse model of heart failure induced by isoproterenol, Fan et al. [29] demonstrated that AMPK activation promotes sirtuin 1 (SIRT1) activation through phosphorylation of PGC-1α and elevation of NAD^+^ levels. Activated SIRT1 subsequently deacetylates PGC-1α, forming an AMPK/SIRT1/PGC-1α positive feedback loop. This loop ultimately upregulates the anti-apoptotic protein Bcl-2, downregulates the pro-apoptotic protein Bax, and inhibits caspase-3 activity, thereby suppressing cardiomyocyte apoptosis. Therefore, targeted modulation of the AMPK pathway may inhibit excessive cardiomyocyte apoptosis and preserve cardiac structure and function in HF.

Alleviating Chronic Myocardial Inflammation. Chronic low-grade inflammation plays a key role in driving HF progression. In contrast to the transient and defensive role of acute inflammation, the persistent inflammatory state in HF involves repeated activation of inflammatory signaling pathways. This persistent inflammatory state is self-perpetuating, driven by continuous leukocyte infiltration and cytokine release. These processes lead to myocardial fibrosis, cellular apoptosis, and adverse ventricular remodeling, collectively contributing to the transition from compensated cardiac function to overt decompensation [30]. AMPK exerts significant anti-inflammatory and cardioprotective effects, in part through its modulation of key inflammatory signaling pathways. Nuclear factor kappa B (NF-κB) is a central transcription factor that regulates inflammatory responses. Its activation depends on the degradation of its inhibitory protein, IκBα, which allows NF-κB to translocate to the nucleus and drive the expression of pro-inflammatory cytokines such as tumor necrosis factor-alpha (TNF-α) and interleukin-6 (IL-6) [31]. Studies have demonstrated that AMPK, potentially through mechanisms involving PPARα, can inhibit IκBα degradation and subsequent NF-κB nuclear translocation, thereby suppressing the transcriptional production of key pro-inflammatory mediators [32]. Thus, by attenuating NF-κB signaling, AMPK activation may help mitigate the chronic inflammatory response that drives HF progression.

Inhibiting the Progression of Myocardial Hypertrophy. Myocardial hypertrophy initially represents an adaptive response of the heart to sustained pressure or volume overload. While this compensatory mechanism helps maintain cardiac output by augmenting contractility, prolonged and excessive hypertrophy becomes maladaptive. It triggers a cascade of pathological sequelae—including dysregulated energy metabolism and systolic and diastolic dysfunction—that ultimately culminates in HF [33]. Energy metabolic imbalance is a hallmark of pathological hypertrophy. AMPK regulates mitochondrial function and energy metabolism by activating PGC-1α, which enhances the expression and activity of key enzymes involved in fatty acid oxidation while suppressing non-productive glycolytic pathways [34]. These actions help correct the abnormal energy substrate preference in hypertrophic cardiomyocytes, thereby reducing cellular energy metabolic load. Furthermore, the mechanistic target of rapamycin complex 1 (mTORC1) is a well-established driver of pathological hypertrophy through its promotion of protein synthesis. Li et al. [35] reported that in a pressure overload-induced heart failure mouse model, activation of AMPK with 5-aminoimidazole-4-carboxamide ribonucleotide (AICAR) inhibited the activity of mechanistic target of rapamycin complex 1 (mTORC1) and its downstream effector p70 ribosomal S6 kinase (p70S6K), thereby suppressing cardiac hypertrophy progression. Therefore, modulating the AMPK pathway may represent an approach to decelerate the transition from compensatory myocardial hypertrophy to overt heart failure.

Regulating Myocardial Autophagy Homeostasis. Autophagy is a conserved lysosome-dependent process that degrades abnormal proteins and damaged organelles. It plays a vital role in maintaining cardiomyocyte homeostasis and facilitating the recycling of cellular components and energy [36]. Under physiological conditions, basal autophagy ensures the timely clearance of dysfunctional mitochondria and toxic metabolites, thereby supporting mitochondrial integrity and metabolic efficiency in the heart. However, pathological stresses such as ischemia, hypoxia, or pressure overload can disrupt autophagic flux, leading to either excessive or insufficient activation. This dysregulation contributes to myocardial dysfunction and accelerates the progression of HF [37,38]. Upon activation, AMPK regulates autophagy through multiple intersecting pathways. Cardiac aging is a major risk factor for heart failure. The aged heart exhibits heightened stress susceptibility, impaired autophagic flux, and progressive mitochondrial dysfunction, all of which are hallmark features of the failing heart [39]. Within this context, AMPK regulates autophagy through conserved mechanisms. It directly phosphorylates ULK1 at Ser317 and Ser777 to promote autophagosome formation, thereby counteracting mTORC1-mediated inhibitory phosphorylation of ULK1 at Ser757 [40]. First characterized in cardiac aging, this regulatory axis is likely to contribute to autophagy regulation in the failing heart. Additionally, AMPK suppresses mTORC1 activity by phosphorylating its regulatory components, such as the tuberous sclerosis complex 2 (TSC2) protein, thereby promoting autophagic initiation, as demonstrated by Wang et al. in a post-acute myocardial infarction heart failure model [41]. Notably, the cardiac-predominant AMPKα2 isoform plays a specific role in regulating mitochondrial quality control. Studies have demonstrated that in failing human hearts and pressure-overloaded mouse models, overexpression of AMPKα2 promotes mitophagy, the selective autophagy of mitochondria. This occurs via phosphorylation of PTEN-induced kinase 1 (PINK1) at Ser495, which facilitates the recruitment of the E3 ubiquitin ligase Parkin. The enhanced PINK1-Parkin-SQSTM1/p62 axis promotes the clearance of damaged mitochondria, improves mitochondrial function, reduces apoptosis, and delays HF progression [42]. In summary, AMPK helps restore myocardial autophagy homeostasis, thereby protecting the failing heart.

Beyond the mechanisms detailed above, AMPK participates in HF progression through other molecular pathways, including the regulation of ferroptosis, microRNA (miRNA) networks, and the PRL2-AMPKα2 axis. Ferroptosis is a form of regulated cell death driven by iron-dependent lipid peroxidation and has been implicated in cardiomyocyte loss during HF. Preclinical studies have demonstrated that AMPK activation mitigates ferroptotic injury by upregulating key anti-ferroptotic proteins such as glutathione peroxidase 4 (GPX4) and ferroptosis suppressor protein 1 (FSP1), thereby inhibiting excessive ROS generation and preserving mitochondrial homeostasis [43,44]. MicroRNAs are small non-coding RNAs that fine-tune gene expression post-transcriptionally and are involved in diverse cardiac processes. In a myocardial infarction-induced HF model, the exercise-induced metabolite β-aminoisobutyric acid (BAIBA) was shown to upregulate miR-208b, which subsequently promoted AMPK phosphorylation. This miR-208b/AMPK axis reduced the expression of pro-apoptotic proteins, enhanced ATP production, and ameliorated mitochondrial dysfunction in failing cardiomyocytes, effects that were abolished by AMPK inhibition [45]. Recent studies have identified phosphatase of regenerating liver 2 (PRL2) as a negative regulator of cardiac AMPKα2. Upregulated in human and experimental HF, PRL2 directly dephosphorylates Thr172 via its catalytic site (C46) to suppress AMPK activity, promoting mitochondrial dysfunction and aggravated cardiac hypertrophy. Conversely, myocardial-specific PRL2 knockdown restores AMPKα2 phosphorylation and attenuates adverse remodeling [8].

### 3.4. Traditional Chinese Medicine Regulating the AMPK Signaling Pathway for Treating Heart Failure

Although the term “heart failure” does not appear explicitly in classical TCM texts, clinical presentations characteristic of the condition have been documented for centuries. The pathogenesis and progression of HF correlate closely with TCM syndromes such as “decline of heart qi,” “insufficiency of heart yang,” and the “intermingling of phlegm, blood stasis, and water retention” [46]. For instance, The Yellow Emperor’s Inner Canon: Basic Questions states, “In heart obstruction, the vessels are not free-flowing,” an early observation of impaired cardiac circulation. It also notes, “If one cannot lie down and experiences panting when lying flat, it is due to the lodging of water qi”—a description highly consistent with the orthopnea and paroxysmal nocturnal dyspnea seen in HF. The Treatise on Febrile Diseases describes “palpitations with an irregular pulse,” indicative of cardiac arrhythmia, and introduces classic formulas such as Zhigancao Decoction. Furthermore, Synopsis of Prescriptions of the Golden Chamber records, “In heart water disease, the body feels heavy with shortness of breath, inability to lie down, restlessness and agitation, and swelling of the lower body,” a syndrome strikingly similar to modern HF, and establishes corresponding therapeutic principles, such as warming yang to promote diuresis.

#### 3.4.1. Active Components of Traditional Chinese Medicine

**Figure 4:**
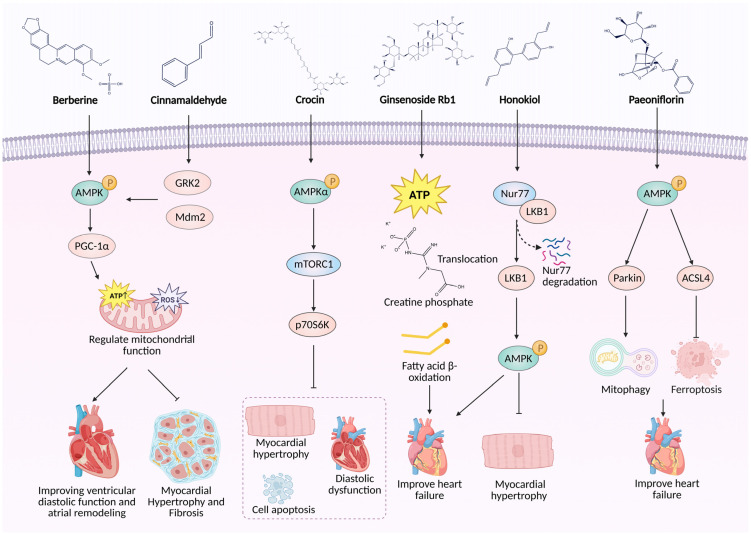
Schematic illustration of TCM bioactive components modulating the AMPK pathway in heart failure. For detailed description, see Section 3.4.1. Created in BioRender. Peng, S. (2026) https://BioRender.com/0u9olfy (accessed on 13 March 2026).

Berberine, an isoquinoline alkaloid from Coptis chinensis, improves diastolic function and attenuates atrial remodeling in a mouse model of HFpEF through AMPK activation, leading to upregulated PGC-1α and enhanced mitochondrial biogenesis [47]. Molecular docking predicted direct binding of berberine to AMPK at ASN-150, and AMPKα1/α2 siRNA abolished its protective effects in H9C2 cells, confirming AMPK dependency. Similarly, cinnamaldehyde, a phenylpropanoid from cinnamon, targets GRK2 to promote its Mdm2-mediated ubiquitination and degradation, thereby activating the AMPK/PGC-1α axis and ameliorating isoproterenol-induced heart failure in rats. GRK2 overexpression attenuated these effects, establishing GRK2 as a key upstream mediator of AMPK activation [48]. Crocin, a carotenoid from saffron, alleviates angiotensin II-induced cardiac hypertrophy and apoptosis by activating AMPKα and inhibiting the downstream mTOR/p70S6K pathway. These effects were reversible by AMPK blockade with Compound C, indicating that AMPK activation is essential for crocin’s actions [49]. In another line of evidence, ginsenoside Rb1, a major ginsenoside from Panax ginseng, increases myocardial high-energy phosphates and enhances fatty acid β-oxidation in an AMPK-dependent manner in adriamycin-induced chronic heart failure rats, with the AMPK inhibitor Ara A abolishing these effects and the AMPK activator Aicar mimicking them, thereby confirming pathway involvement [50]. Honokiol, a lignan from Magnolia officinalis, suppresses pathological hypertrophy and heart failure progression by promoting dissociation of the Nur77-LKB1 complex, leading to LKB1 nuclear export and subsequent AMPK activation; co-immunoprecipitation studies confirmed Nur77-LKB1 dissociation as the initiating event, and Nur77 overexpression reversed the cardioprotective effects [51]. Furthermore, paeoniflorin, a monoterpene glycoside from peony root, alleviates isoproterenol-induced cardiac hypertrophy via dual AMPK-mediated mechanisms: it initiates Parkin-dependent mitophagy and inhibits ACSL4-mediated ferroptosis. CETSA confirmed direct binding to AMPKα2, and Compound C reversed its protective effects, thereby establishing a direct AMPK-targeting mechanism [52]. Figure 4 illustrates the diverse AMPK-dependent pathways through which these TCM components exert their anti-heart failure effects. A summary of these active ingredients is presented in Table 1.

**Table 1:** Active components of Traditional Chinese Medicine targeting the AMPK signaling pathway in HF.

TCM Active Ingredient	Chemical Formula	Model System	HF Type	Pathway	Key Endpoints	Ref.
Berberine	C_20_H_18_NO_4_^+^	C57BL/6J mice (100, 200 mg/kg/d); H9C2 cells (20 μM)	HFpEF	AMPK/PGC-1α	Improved cardiac function and ventricular diastolic function; attenuated myocardial hypertrophy, fibrosis, and apoptosis; enhanced mitochondrial function and energy metabolism	[47]
Cinnamaldehyde	C_9_H_8_O	SD rats (20, 40, 80 mg/kg/d); Primary neonatal rat cardiomyocytes (NRCMs) (10, 20, 40 μM)	ISO-induced HF	GRK2/AMPK/PGC-1α	Improved cardiac function; attenuated myocardial injury, fibrosis and hypertrophy; enhanced fatty acid metabolism and mitochondrial function; activated AMPK/PGC-1α pathway via GRK2 degradation	[48]
Crocin	C_44_H_64_O_24_	SD rats (40 mg/kg/d); H9C2 cells (10, 20, 40 μM)	Ang II-induced cardiac hypertrophy	AMPKα/mTOR/p70S6K	Reduced blood pressure; improved cardiac function; attenuated cardiac hypertrophy and apoptosis; activated AMPKα and inhibited mTOR/p70S6K signaling	[49]
Ginsenoside Rb1	C_54_H_92_O_23_	Wistar rats (10 mg/100 g)	Adriamycin-induced chronic HF	AMPK	Improved cardiac function; enhanced myocardial energy metabolism; increased high-energy phosphates and FAO enzyme activity; modulated L-carnitine and malonyl-CoA levels	[50]
Honokiol	C_18_H_18_O_2_	SD rats (2.5, 5 mg/kg/d); Neonatal rat ventricular myocytes (NRVMs) (10^−8^ mol/L)	Ang II-induced cardiac hypertrophy	Nur77-LKB1-AMPK	Attenuated myocardial hypertrophy, fibrosis and dysfunction; reduced cardiomyocyte apoptosis; promoted dissociation of Nur77-LKB1 complex and activation of AMPK signaling	[51]
Paeoniflorin	C_23_H_28_O_11_	C57BL/6J mice (10, 20, 40 mg/kg/d); H9c2 cells (400 μM)	ISO-induced cardiac hypertrophy	AMPK-Parkin-ACSL4	Improved cardiac function; attenuated myocardial hypertrophy and fibrosis; reduced oxidative stress and ferroptosis; enhanced mitophagy and mitochondrial energy metabolism via AMPK/Parkin pathway	[52]

#### 3.4.2. Herbal Formulas of Traditional Chinese Medicine

Kanli Granule, a formula comprising ten herbs including Aconiti Lateralis Radix Praeparata (Fuzi), Astragali Radix (Huangqi), and Poria (Fuling), is clinically used for chronic heart failure with heart-kidney yang deficiency and internal retention of fluid and blood stasis. Zhao et al. employed network pharmacology and molecular docking to predict that its cardioprotective effects involve regulation of fatty acid metabolism, with PPARα and CPT1A as core targets [20]. These predictions were validated in a rat model of abdominal aortic constriction-induced heart failure, where Kanli Granule activated the myocardial AMPK/PPARα/CPT1A pathway, enhanced fatty acid β-oxidation, increased the myocardial ATP/ADP ratio, and preserved cardiac function.

Fuyu Decoction, composed of *Corni Fructus* (Shanzhuyu), *Aconiti Lateralis Radix Praeparata* (Fuzi), *Glycyrrhizae Radix* (Gancao), is clinically used to supplement qi, warm yang, resolve stasis, and unblock collaterals. In a rat model of HF induced by coronary artery ligation, Fuyu Decoction significantly improved cardiac function and attenuated ventricular remodeling. Mechanistically, it downregulated myocardial p-AMPK, LC3-II, and Beclin-1 expression while upregulating p-mTOR and p62, indicating inhibition of excessive autophagy. These effects were abrogated by the AMPK agonist EX229, confirming that Fuyu Decoction confers cardioprotection through suppression of AMPK/mTOR pathway-mediated autophagy [53]. It is worth noting that the role of AMPK in myocardial autophagy is context-dependent. Basal AMPK activity maintains autophagic flux for cellular homeostasis, whereas sustained AMPK activation under prolonged stress may drive excessive autophagy and exacerbate ventricular remodeling. Thus, the opposing effects of AMPK modulation may reflect different pathological drivers at various stages or types of HF.

Linggui Zhugan Decoction, originating from Zhang Zhongjing’s Treatise on Cold Damage Diseases and consisting of Poria (Fuling), Cinnamomi Ramulus (Guizhi), Atractylodis Macrocephalae Rhizoma (Baizhu), and Glycyrrhizae Radix (Gancao), functions to warm yang, resolve fluid retention, fortify the spleen, and drain dampness. It improves cardiac function and restores mitochondrial membrane potential in post-myocardial infarction chronic heart failure rats [54]. These effects were accompanied by upregulated SIRT1, p-AMPK, PGC-1α, NRF1, and TFAM, indicating activation of the SIRT1/AMPK/PGC-1α pathway.

Wenxin Keli, a Chinese patent medicine for heart failure-related arrhythmias, restores mitochondrial oxidative phosphorylation and reduces arrhythmia susceptibility in post-myocardial infarction heart failure rats by activating the AMPK/SIRT1/PGC-1α axis [55]. This activation enhances the interaction between PGC-1α and its transcriptional coactivators PPARs and ERRs, orchestrating fatty acid β-oxidation and glucose utilization, leading to elevated myocardial ATP content and improved connexin 43 phosphorylation.

Xinbao Pill, derived from “Liushen Pill” and “Shenfu Decoction,” attenuates cardiac hypertrophy, fibrosis, and dysfunction in isoproterenol-induced HF models by suppressing sodium-glucose cotransporter 1 (SGLT1) expression. This inhibition enhances AMPK phosphorylation, promoting PPARα nuclear translocation and transcriptional activity, which upregulates fatty acid oxidation enzymes, restores mitochondrial function, and improves myocardial energy metabolism. The causal role of SGLT1 was validated by loss- and gain-of-function experiments: SGLT1 knockdown potentiated, whereas its overexpression abolished, the cardioprotective effects of Xinbao Pill on cardiac energy metabolism [56].

**Figure 5:**
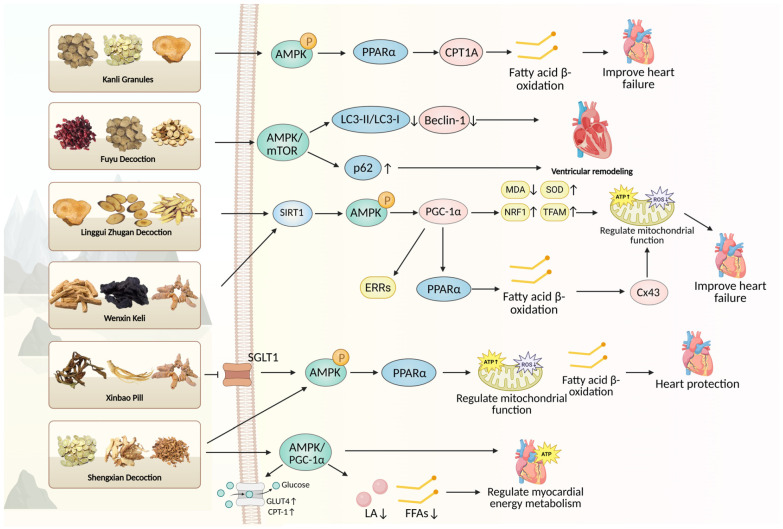
Schematic illustration of TCM herbal formulas targeting the AMPK pathway in heart failure. For detailed description, see Section 3.4.2. Created in BioRender. Peng, S. (2026) https://BioRender.com/0u9olfy (accessed on 13 March 2026).

Shengxian Decoction, a classic formula from Zhang Xichun’s Records of Traditional Chinese and Western Medicine in Combination comprising *Astragali Radix* (Huangqi), *Anemarrhenae Rhizoma* (Zhimu), *Bupleuri Radix* (Chaihu), *Platycodonis Radix* (Jiegeng), and *Cimicifugae Rhizoma* (Shengma), improves cardiac function, attenuates myocardial apoptosis and oxidative stress, and restores mitochondrial integrity in transverse aortic constriction-induced chronic heart failure rats [57]. These effects were associated with increased AMPK phosphorylation and upregulated expression of PGC-1α, NRF1, TFAM, CPT-1, and GLUT4, indicating enhanced mitochondrial biogenesis and metabolic flexibility. Figure 5 illustrates the AMPK-dependent pathways through which these TCM formulas exert their anti-heart failure effects. A summary of these formulas is presented in Table 2.

**Table 2:** TCM formulas targeting the AMPK signaling pathway in heart failure.

TCM Formulas Treat	Composition	Model System	HF Type	Pathway	Key Endpoints	Ref.
Kanli Granule	*Aconiti Lateralis Radix Praeparata* (Fuzi), *Astragali Radix* (Huangqi), *Poria* (Fuling), *Atractylodis Macrocephalae Rhizoma*(Baizhu), *Paeoniae Radix Alba* (Baishao), *Plantaginis Semen* (Cheqianzi), *Curcumae Rhizoma* (Ezhu), *Ophiopogonis Radix*(Maidong), *Sparganii Rhizoma* (Sanleng), *Lepidii Semen* (Tinglizi)	Wistar rats (0.675, 1.35, 2.7 g/kg)	AAC-induced chronic HF	AMPK/PPARα/CPT1A	Improved cardiac function; attenuated myocardial hypertrophy and fibrosis; enhanced fatty acid metabolism and myocardial energy metabolism; activated AMPK/PPARα/CPT1A pathway	[20]
Fuyu Decoction	*Corni Fructus* (Shanzhuyu), *Aconiti Lateralis Radix Praeparata* (Fuzi), *Glycyrrhizae Radix* (Gancao)	Wistar rats (5.0 g/kg)	CAL-induced HF	AMPK/mTOR	Improved cardiac function; attenuated ventricular remodeling and myocardial infarction area; inhibited cardiomyocyte apoptosis; suppressed excessive autophagy via AMPK/mTOR pathway inhibition	[53]
Linggui Zhugan Decoction	*Poria* (Fuling), *Cinnamomi Ramulus* (Guizhi), *Atractylodis Macrocephalae Rhizoma* (Baizhu), *Glycyrrhizae Radix* (Gancao)	SD rats (5.4 g/kg/d)	Post-MI chronic HF	SIRT1/AMPK/PGC-1α	Improved cardiac function and exercise tolerance; attenuated oxidative stress and myocardial injury; restored mitochondrial function and membrane potential; activated SIRT1/AMPK/PGC-1α pathway	[54]
Wenxin Keli	*Codonopsis Radix* (Dangshen), *Polygonati Rhizoma* (Huangjing), *Notoginseng Radix et Rhizoma* (Sanqi), *Succinum* (Hupo), *Nardostachyos Radix et Rhizoma* (Gansong)	SD rats (2.7 g/kg)	Post-MI HF	AMPK/SIRT1/PGC-1α	Improved cardiac function; attenuated myocardial fibrosis and hypertrophy; enhanced mitochondrial function and energy metabolism; reduced arrhythmia susceptibility; activated AMPK/SIRT1/PGC-1α pathway	[55]
Xinbao Pill	*Daturae Flos* (Yangjinhua), *Ginseng Radix et Rhizoma* (Renshen), *Notoginseng Radix et Rhizoma* (Sanqi), *Cinnamomi Cortex*(Rougui), *Aconiti Lateralis Radix Praeparata*(Fuzi), *Cervi Cornu Pantotrichum* (Lurong), *Bufonis Venenum* (Chansu), *Borneolum Syntheticum* (Bingpian), *Moschus* (Shexiang)	SD rats (60, 90, 120 mg/kg/d); Neonatal rat ventricular myocytes (NRVMs) (8, 16, 32 μg/μL)	ISO-induced HF	SGLT1/AMPK/PPARα	Improved cardiac function; attenuated myocardial hypertrophy and fibrosis; enhanced fatty acid metabolism and mitochondrial function; suppressed SGLT1 expression; activated AMPK/PPARα pathway	[56]
Shengxian Decoction	*Astragali Radix* (Huangqi), *Anemarrhenae Rhizoma* (Zhimu), *Bupleuri Radix* (Chaihu), *Platycodonis Radix* (Jiegeng), *Cimicifugae Rhizoma* (Shengma)	SD rats (5.1, 10.2, 20.4 g/kg/d)	TAC-induced CHF	AMPK/PGC-1α	Improved cardiac function; attenuated myocardial apoptosis and oxidative stress; restored mitochondrial structure and function; enhanced glucose and lipid metabolism; activated AMPK/PGC-1α pathway	[57]

### 3.5. Evidence Levels of TCM Interventions

Table 3 summarizes the evidence levels for all included TCM interventions based on the validation approaches employed. Among active components, cinnamaldehyde and paeoniflorin showed direct target engagement confirmed by CETSA and loss-of-function rescue [48,52]. Berberine, crocin, ginsenoside Rb1, and honokiol demonstrated pathway-specific effects validated by pharmacological inhibitors or genetic approaches [47,49,50,51]. For most complex herbal formulas, AMPK pathway activation was observed but causality was not established through loss-of-function studies [19,53,54,55,56]. Fuyu Decoction was an exception where AMPK agonist EX229 confirmed pathway-specific involvement [52].

**Table 3:** Evidence levels of TCM interventions targeting the AMPK pathway in HF.

TCM Active Ingredient and Formulas Treat	Direct AMPK Mechanisms	Correlative Evidence	Hypothesized/Unproven Mechanisms	Ref.
Berberine	Molecular docking confirmed binding to AMPK at ASN-150; AMPKα1/α2 siRNA abolished protective effects in H9C2 cells	Western blot showed increased p-AMPK, PGC-1α, NRF1, TFAM; TUNEL and JC-1 staining demonstrated reduced apoptosis and improved mitochondrial membrane potential	Gut microbiota modulation as a contributor to cardioprotection in HFpEF models lacks validation; direct binding to other AMPK isoforms (α1) remains unconfirmed	[47]
Cinnamaldehyde	CETSA and MST confirmed direct binding to GRK2; GRK2 overexpression reversed the effects on AMPK/PGC-1α and fatty acid metabolism; promoted Mdm2-mediated GRK2 ubiquitination	Western blot showed increased p-AMPK, PGC-1α, ACADM, CPT-1B, CD36, NRF-1; JC-1 and Mito-tracker staining indicated restored mitochondrial membrane potential	The involvement of other E3 ubiquitin ligases in GRK2 degradation has not been established; potential GRK2-independent AMPK activation remains unexplored	[48]
Crocin	AMPK inhibitor Compound C abolished anti-hypertrophic and anti-apoptotic effects in H9C2 cells	Western blot showed increased p-AMPKα, decreased p-mTOR and p-p70S6K; RT-qPCR revealed reduced ANP and β-MHC mRNA; TUNEL staining showed decreased apoptosis	Direct binding to AMPK has not been demonstrated by molecular docking or CETSA; contribution of other upstream kinases (e.g., LKB1, CaMKKβ) cannot be ruled out	[49]
Ginsenoside Rb1	AMPK inhibitor Ara A abolished, and AMPK activator Aicar mimicked the effects on FAO and energy metabolism in failing heart	HPLC showed increased PCr, ATP, ADP, PCr/ATP ratio; ELISA demonstrated elevated free L-carnitine and malonyl-CoA; enzyme activity assays showed upregulated CPT-1, MCAD, ACSL	PI3K/Akt pathway involvement in energy metabolism regulation (suggested by prior studies) lacks experimental validation; upstream AMPKs remain to be identified	[50]
Honokiol	Promoted dissociation of Nur77-LKB1 complex (co-IP), leading to LKB1 nuclear export and AMPK activation; Nur77 overexpression reversed these effects	Western blot showed increased p-LKB1, p-AMPK, decreased p-p70S6K; immunofluorescence demonstrated LKB1 translocation to cytoplasm; RT-qPCR showed reduced Nur77 mRNA	Potential regulation of other NR4A family members (Nurr1, NOR-1) has not been investigated; the specific E3 ligase mediating Nur77 ubiquitination remains unidentified	[51]
Paeoniflorin	CETSA confirmed binding to AMPKα2; molecular docking predicted binding energy of −8.5 kcal/mol; Compound C and Parkin siRNA reversed protective effects on ferroptosis and mitophagy	Western blot showed increased p-AMPK, Parkin, PINK1, decreased mitochondrial ACSL4; flow cytometry revealed reduced ROS and Fe^2+^; lipidomics identified seven differentially regulated lipid metabolites	Involvement of other mitophagy pathways (FUNDC1, BNIP3) has not been validated; Nrf2 as a downstream AMPK effector in antioxidant effects remains unconfirmed	[52]
Kanli Granule	Molecular docking predicted strong binding affinities between key components (oleanolic acid, hederagenin) and PPARα/CPT1A	Western blot and qPCR showed upregulated AMPK, PPARα, CPT1A expression; increased ATP/ADP ratio and reduced FFA levels; improved cardiac function and myocardial structure	Network pharmacology predictions of additional targets (SREBF1, PPARG, FASN) lack experimental validation; potential involvement of other metabolic pathways remains unexplored	[20]
Fuyu Decoction	AMPK agonist EX229 reversed the protective effects, confirming pathway-specific involvement	Western blot showed downregulated p-AMPK, LC3-II, Beclin-1; upregulated p-mTOR, p62; improved cardiac function and reduced myocardial infarction area	Involvement of other autophagy pathways (e.g., PI3K/Akt) has not been established; potential direct interaction with AMPK or mTOR remains unconfirmed	[53]
Linggui Zhugan Decoction	No direct validation (e.g., inhibitor or siRNA studies)	Western blot and qPCR showed upregulated SIRT1, p-AMPK, PGC-1α, NRF1, TFAM; JC-1 staining showed restored mitochondrial membrane potential; TEM demonstrated improved mitochondrial ultrastructure; reduced MDA and increased SOD	The specific active components responsible for SIRT1/AMPK activation have not been identified; involvement of other energy metabolism pathways (e.g., PPARs) remains unexplored	[54]
Wenxin Keli	No direct validation (e.g., inhibitor or siRNA studies)	Western blot showed upregulated ATP5D, Cx43, p-Cx43; increased mitochondrial complex activities and ATP content; improved cardiac function and reduced arrhythmia susceptibility	Direct phosphorylation of downstream targets by AMPK or mediation through intermediate kinases remains unclear; involvement of other signaling pathways (e.g., PI3K/Akt) cannot be excluded	[55]
Xinbao Pill	SGLT1 knockdown potentiated, while overexpression abolished the cardioprotective effects; confirmed SGLT1 as a critical upstream target	Western blot showed downregulated SGLT1, upregulated p-AMPK, PPARα, FAO enzymes (CD36, CPT-1B, ACADM); improved cardiac function and mitochondrial integrity	Involvement of other glucose transporters (e.g., SGLT2, GLUT4) has not been established; direct binding of components to SGLT1 or AMPK lacks validation by molecular docking or CETSA	[56]
Shengxian Decoction	No direct validation (e.g., inhibitor or siRNA studies)	Western blot showed upregulated p-AMPK, PGC-1α, NRF1, TFAM, CPT-1, GLUT4; increased ATP and ATP5D; improved cardiac function and reduced oxidative stress	Upstream or downstream relationship between AMPK and PGC-1α remains unclear; involvement of other mitochondrial biogenesis regulators (e.g., ERRα, PPARα) has not been investigated	[57]

### 3.6. Safety and Standardization Considerations for TCM

Despite the promising preclinical evidence discussed above, several factors should be considered when interpreting these findings and translating them into clinical practice. Batch-to-batch variability in herbal material quality due to differences in cultivation and processing remains a significant challenge for standardization. The potential toxicity of certain herbs, particularly those containing alkaloids (e.g., *Aconiti Lateralis Radix Praeparata*), warrants systematic long-term safety evaluation. Additionally, the risk of herb-drug interactions in patients receiving concomitant guideline-directed medical therapy remains largely unexplored. Addressing these issues through rigorous quality control and well-designed pharmacokinetic studies will be essential for the safe and effective application of TCM in HF management.

## 4. Discussion

### 4.1. Principal Findings

This systematic review provides a comprehensive synthesis of preclinical evidence on TCM-mediated AMPK modulation in heart failure. The principal findings are (1) direct evidence for AMPK-dependent cardioprotection remains limited to a small subset of TCM interventions, with cinnamaldehyde and paeoniflorin representing the most rigorously validated examples; (2) several active compounds demonstrate pathway-specific effects validated by pharmacological or genetic approaches, though questions regarding upstream regulators or parallel pathways remain; (3) for most complex herbal formulas, evidence is largely correlative, with causality unconfirmed; (4) notable methodological limitations, including reliance on H9C2 cells and acute injury models, temper translational potential.

### 4.2. Comparison with Previous Reviews

Unlike previous narrative reviews that separately addressed AMPK in HF pathogenesis or general TCM cardioprotection, this systematic review provides an integrated, evidence-stratified perspective. Our findings extend prior work by systematically applying predefined criteria to distinguish direct AMPK mechanisms from correlative evidence, identifying specific interventions with the most rigorous validation, and delineating research gaps that must be addressed to advance the field.

### 4.3. Mechanistic Insights and Evidence Strength

The spectrum of mechanistic evidence revealed in this review highlights important considerations for future research. Direct target engagement studies, such as CETSA for paeoniflorin [52] and GRK2 binding assays for cinnamaldehyde [48], provide the most reliable evidence for AMPK-dependent mechanisms. Studies employing multiple validation approaches—combining molecular docking, pharmacological inhibitors, and genetic manipulation—consistently provided the most robust mechanistic insights [47,48,52]. In contrast, studies relying solely on changes in phosphorylated AMPK without pathway-specific validation [54,57] leave causality unconfirmed and should be interpreted with caution.

### 4.4. Methodological Limitations and Translational Relevance

Several methodological limitations in the included studies temper the translational potential of the findings. First, the predominance of H9C2 cardiomyoblast cells in mechanistic studies raises concerns about physiological relevance, as these cells lack the mature contractile and metabolic features of adult cardiomyocytes. Second, the reliance on acute, toxin-induced injury models (isoproterenol, adriamycin) may not adequately recapitulate the complex pathophysiology of chronic heart failure in aging populations with comorbidities. Third, the absence of systematic off-target controls leaves open the possibility that observed effects are mediated partially through AMPK-independent pathways.

### 4.5. Research Gaps and Future Directions

Based on this analysis, several priority areas emerge: (1) Causality validation: Future studies should incorporate genetic models (cardiomyocyte-specific AMPKα2 knockout) and rigorous pharmacological approaches (selective AMPK inhibitors with appropriate off-target controls) to establish causal relationships, particularly for complex herbal formulas where active components are less defined. (2) Model refinement: Transition from H9C2 cells to human induced pluripotent stem cell-derived cardiomyocytes (iPSC-CMs) and cardiac organoids would enhance physiological relevance. Where feasible, confirmation in human failing heart samples is warranted. (3) Context-aware design: Given the context-dependent role of AMPK in autophagy and other processes, studies should explicitly examine how effects differ across HF stages (compensated hypertrophy vs. decompensated failure) and etiologies (HFrEF vs. HFpEF). (4) Deconvolution of complex formulas: Integration of network pharmacology with bioassay-guided fractionation and systematic loss-of-function studies is needed to identify active compounds and their precise molecular targets within multi-herb formulations. (5) Translation-focused endpoints: Beyond molecular markers, studies should assess functional outcomes relevant to clinical practice, including exercise tolerance, quality of life, and long-term survival in appropriate animal models. (6) Safety and interactions: Systematic evaluation of herb-drug interactions with guideline-directed HF therapies, as well as long-term safety of TCM interventions, particularly those containing potentially toxic herbs, is essential for clinical translation.

### 4.6. Limitations of This Review

This systematic review has several limitations. First, the search was limited to PubMed and Web of Science, potentially omitting relevant studies from other databases. Second, only English-language articles were included, introducing possible language bias. Third, the heterogeneity of experimental models and outcome measures precluded meta-analysis. Fourth, the quality of included studies varied considerably, and we did not perform formal risk of bias assessment using tools such as SYRCLE’s RoB tool, though our evidence-level stratification partially addresses this.

## 5. Conclusions

The current evidence for TCM-mediated AMPK modulation in heart failure remains predominantly preliminary and correlative. Future research should prioritize rigorous mechanistic validation using genetic models, human-relevant systems, and systematic deconvolution of complex formulas to establish causality and advance clinical translation.

## Data Availability

No new data were created or analyzed in this study. Data sharing is not applicable to this article.

## Supplementary Materials

The following supporting information can be downloaded at: https://www.mdpi.com/article/10.3390/biomedicines14040765/s1, Table S1: PRISMA 2020 Checklist.
